# Development and Optimization of an Aminooxy Coupling Reaction to Prepare Multivalent Bioconjugates with a Single Noncanonical Amino Acid

**DOI:** 10.1021/acs.bioconjchem.5c00517

**Published:** 2026-01-20

**Authors:** Robert K. Gourdie, Emily L. Boyt, Brian M. Flood, Alexander C. Williard, William I. Eisen, Tyler L. Skeen, Annalee R. Hassler, Aaron S. Wang, Cedrick R. Dimaranan, Sophia K. Rothman, Elizabeth A. King, Jonathan C. Maza, Douglas D. Young

**Affiliations:** † Department of Chemistry, 8604 William & Mary, Williamsburg, Virginia 23185, United States; ‡ Department of Chemistry, 1438 University of California, Berkeley, Berkeley, California 94720, United States; § Department of Chemistry, 8785 University of California, San Francisco, San Francisco, California 94143, United States

## Abstract

Bioconjugates have increasing utility in numerous medical and materials applications; thus, the development of new mechanisms to increase their valency and functional potential has the ability to further their impact. Expansion of the chemical tools used to prepare bioconjugates affords greater flexibility in their preparation and can improve their potency and specificity. This research integrates genetic code expansion methodologies with bioorthogonal reaction development to prepare homogeneous multivalent bioconjugates. Specifically, a novel bioorthogonal reaction has been optimized, reacting an *O*-alkoxylamine with a 1,3-diyne in the absence of any additional reagents. This reaction has been found to progress to near completion in under 30 min and generate highly stable bioconjugates. Utilizing a cascade sequence involving a bioorthogonal Glaser–Hay coupling, followed by treatment with an aminooxy partner, provides a mechanism to introduce two novel functionalities into proteins. Moreover, the precise control of genetically incorporating an alkynyl amino acid at a specific residue provides a high degree of control over the conjugate structure and activity. This cascade reaction was also optimized to occur in a one-pot fashion, obviating the need for conjugate purification between reactions. Finally, this strategy was employed in producing a highly effective antibody–drug conjugate (ADC) functionalized with monomethyl auristatin E (MMAE) and a fluorescent probe, allowing for monitoring of therapeutic delivery. When tested against HER2+ cells, this trivalent conjugate was specific, potent, and trackable. As this simple proof-of-concept demonstrates, there is limitless potential for the preparation of other therapeutic and diagnostic bioconjugates using this novel approach.

## Graphical abstract

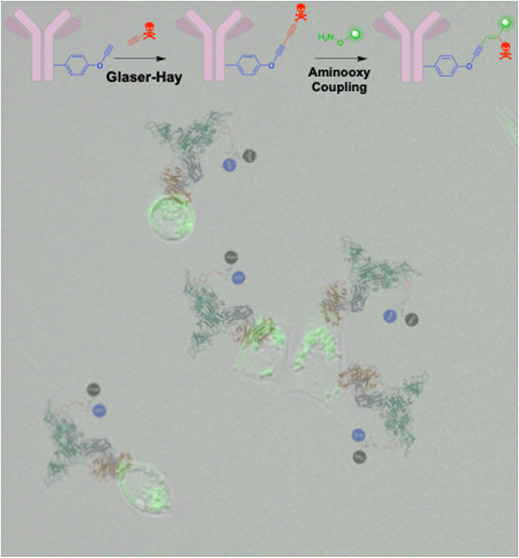

## Introduction

The covalent modification of proteins and other biomolecules, known collectively as bioconjugation, has proven to be a vital tool in drug design, cellular imaging, and diagnostics.^1–4^ Notably, chromatophore- and fluorophore-conjugated antibodies have been used to visualize results in rapid antigen tests for diseases like COVID-19.^5^ Fluorophore–protein conjugates also have numerous applications in biological imaging, both *in vitro* and *in vivo*.^6–9^ With respect to therapeutic value, antibody–drug conjugates (ADCs) have increasing relevance in chemotherapies and other drug delivery applications. ADCs mainly consist of a small-molecule cytotoxic drug covalently linked to a monoclonal antibody.^10–16^ ADCs afford targeted delivery of therapeutics directly to diseased cells without affecting healthy cells, minimizing undesirable off-target effects. Worldwide, over 100 ADCs are in clinical trials, and 14 have been approved for the treatment of various cancers, indicating their therapeutic potential.^10^

Several methods exist for the conjugation of drugs to proteins but are mostly based on the utilization of the nucleophilic groups contained within natural amino acids such as lysine, cysteine, and serine.^2,11,17^ Though bioconjugation to natural amino acid residues is attractive, minimizing the need to bioengineer proteins, it is limited in its selectivity. For example, a typical monoclonal antibody contains around 40 modifiable lysine residues as well as several cysteine residues, some of which may be in the antigen-binding domain.^18–23^ Decreased control over the degree of conjugation translates to a lack of control over the drug dosage being delivered to a tumor. Further, the site of drug conjugation affects the pharmacokinetic profile and stability of an ADC, so nonspecific conjugation introduces another level of uncertainty in the therapeutic value of the conjugate.^24–26^

These issues with conjugation preparation can be circumvented using bioorthogonal chemistries but require the introduction of novel functionality to serve as a reactive handle within the protein.^27–30^ This can be achieved through the genetic engineering of proteins to harbor noncanonical amino acids (ncAAs). Genetic code expansion technologies using degenerate codons or codon expansion are a well-developed field that facilitates the site-specific incorporation of an ncAA into a protein.^31–33^ Numerous ncAAs designed to act as reactive handles have been developed. These ncAAs possess unique chemical moieties that do not cross-react with other biological components, which makes them ideal to be incorporated into proteins for bioorthogonal reactions that occur under physiological conditions.^32^ The most well-known bioorthogonal reactions are the strain-promoted azide–alkyne cycloaddition (SPAAC) and the tetrazine ligation.^34–37^ However, more recently, our lab reported a Glaser–Hay bioconjugation toward the formation of a highly stable and linear carbon–carbon bond.^38–40^ Other reports have utilized ncAA technologies to perform other transition metal-mediated conjugations such as Sonogashira and Suzuki couplings.^41–44^

Examples of using ncAAs in these bioorthogonal reactions toward therapeutic applications are numerous.^31^ However, methods for the site-specific conjugation of multiple small molecules to a protein are extremely limited. The ability to generate a multivalent conjugate is desirable, as an additional function can be granted to the conjugate with different partners. For example, ADCs could be additionally conjugated with a fluorophore or radio-labeled probe, allowing for visualization *in vivo* or *in vitro*. The ADC could also be coupled to a polyethylene glycol (PEG) or affinity tag to increase physiological stability and delivery or allow for more rapid purification of the ADC, respectively.

Examples of multifunctional conjugates do exist, but each method carries with it limitations to its applicability.^45,46^ Hence, site-specific methods for synthesizing multifunctional conjugates require further investigation. These methods have often involved the production of proteins containing multiple different ncAAs, each serving as a handle for a different bioconjugation reaction. This process requires equipping the expression host with multiple orthogonal aminoacyl-tRNA synthase (aaRS)-tRNA pairs, where each recognizes a different codon such as the amber UAG stop codon, the opal UAA stop codon, the ochre UGA stop codon, or a quadruplet codon.^47–52^ In fact, there have even been genetically modified ribosomes developed for the incorporation of ncAAs.^53^ While methods similar to these have been successfully used for site-specific multiconjugation,^54^ the expression of multiple ncAA-containing proteins requires significant additional genetic manipulation of host organisms and results in lower protein yields. Previous work has achieved single-ncAA multivalent conjugates via genetically encoding a bifunctional tetrazine-azide ncAA capable of being labeled by both a tetrazine-(trans-cyclooctene) inverse electron-demand Diels–Alder reaction and a strain-promoted azide–alkyne cycloaddition.^55^ Though this method did benefit from the absence of metal catalysts, it was limited by potential cross-reactivity, as cyclooctynes are known to react with 1,2,4,5-tetrazines in [4 + 2] cycloadditions.^56^

Consequently, we became interested in developing new methodologies for synthesizing multivalent bioconjugates via sequential bioorthogonal couplings from a single ncAA. While we previously reported a cascade synthesis involving a halo-alkyne 1,3-cycloaddition coupled with a Sonogashira coupling,^57^ we hoped to leverage the electron-rich diyne generated through the Glaser–Hay coupling as a starting point for a second reaction. This approach may have several advantages over those previously reported, including the use of fewer transition metal catalysts, improved reaction kinetics, and increased specificity. Moreover, the electron density, stability, and linearity of the diyne generated via a Glaser–Hay coupling provide a fruitful starting point for further conjugation.

One promising reaction involving these 1,3-diyne functionalities is a Cope-type hydroamination.^58–60^ Previous research has demonstrated that such a reaction with a hydroxylamine forms an intermediate that isomerizes to yield a 3,5-disubstituted isoxazole. However, its potential biocompatibility was cause for concern as the reaction required relatively high temperatures (110 °C), copious amounts of base, and nonaqueous solvents. More recently, it has been demonstrated that hydroxylamines and activated alkynes could react under more mild conditions and could even be performed on modified proteins.^61,62^ It is important to note that these biological conditions employed N-substituted hydroxylamines and not more common and commercially available *O*-substituted aminooxy moieties employed in oxime formations. Thus, we became interested in investigating the reactivity of these 1,3-diynes with *O*-substituted alkoxyamine functionalities that cannot undergo subsequent isoxazole isomerization. To the best of our knowledge, this type of reaction has not been reported in the literature; however, initial small-molecule investigations indicated some reaction progression in aqueous media.

## Results and Discussion

### Development and Optimization of Multivalent Bioconjugations

Based on these preliminary results, we prepared bivalent conjugates based on a Glaser–Hay bioorthogonal reaction on a model protein system to demonstrate the proof-of-concept. Initially, green fluorescent protein (GFP-151-*p*PrF) was expressed with a noncanonical *p-*propargyloxyphenylalanine (*p*PrF) at residue 151 on the rigid β-barrel of GFP (Figure 1).^63^ This ncAA contains a terminal alkyne that serves as a reaction handle for the subsequent Glaser–Hay reaction. Following protein purification, the modified GFP was reacted with a biotin alkyne under optimized literature conditions for the Glaser–Hay coupling with CuI/TMEDA.^39^ Given the small mass of the biotin functionalization, resulting in no observable shift on SDS-PAGE, successful conjugation was confirmed via mass spectrometry and incubation with streptavidin derivatized beads and fluorescence microscopy, illustrating GFP binding. Incubation of beads with just GFP-151-*p*PrF resulted in no fluorescence, indicating that nonspecific binding to the streptavidin beads was not occurring (Figure 1).

**Figure 1:**
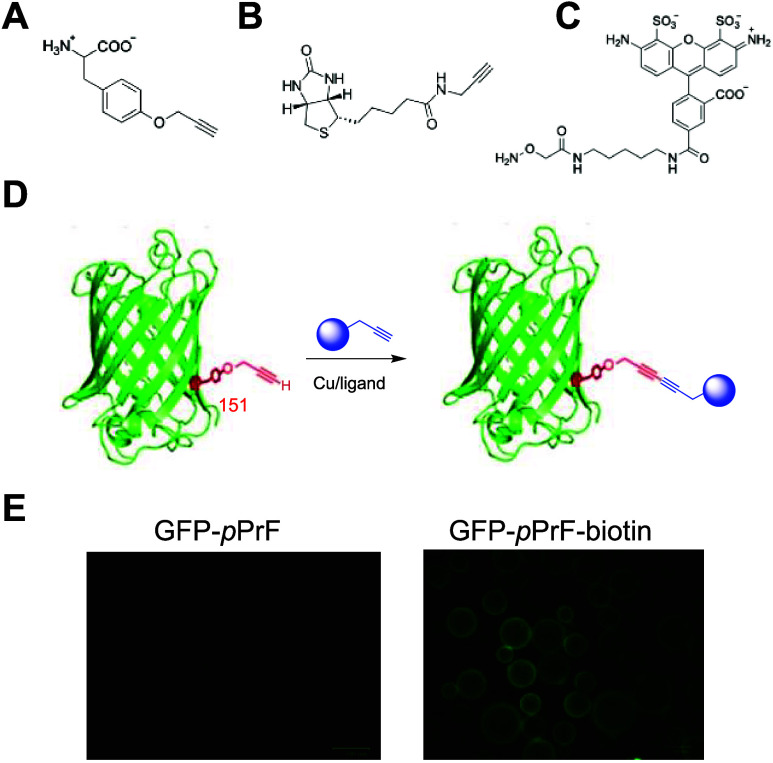
Multivalent bioconjugation components. (A) Alkynyl noncanonical amino acid *p*-propargyloxyphenyalalnine (*p*PrF) structure. (B) Biotin alkyne structure employed in Glaser–Hay bioconjugations. (C) *O*-alkoxylamine fluorophore structure. (D) General scheme for initial ncAA-directed Glaser–Hay bioconjugation to yield a bivalent conjugate. (E) Validation of successful Glaser–Hay coupling. Streptavidin beads were incubated with either GFP harboring an alkynyl ncAA (left) or a bivalent GFP–biotin conjugate (right). After several washes, no GFP fluorescence is observed without the biotin partner, whereas the GFP–Biotin conjugate is immobilized on the bead, resulting in fluorescence.

With a GFP-151-*p*PrF–biotin conjugate in hand, investigations into the reaction with an aminooxy moiety were then explored. For ease of analysis, initial experiments employed a commercially available AlexaFluor-488 aminooxy partner. Incubation of the protein conjugate with this fluorophore for 24 h at 37 °C, followed by washing with molecular weight cutoff spin columns to remove excess fluorophore, afforded the conjugation product, as observed by the appearance of a fluorescent signal on denaturing SDS-PAGE (Figure 2B). Although GFP itself is fluorescent, the denaturation step of SDS-PAGE has been found to eliminate its nascent fluorescence, and thus, the signal is due to covalent attachment of the AlexaFluor-488 probe. This fluorescence was further validated via preparing the ubiquitin-48-*p*PrF-biotin–AlexaFluor conjugate and observing both SDS-PAGE fluorescence and streptavidin bead fluorescence after incubation and washing (Figures 2C and S7). To ensure that exposure to copper did not generate any undesired protein oxidation that could facilitate a more standard oxime formation, both cell lysate and wild-type GFP were subjected to the reaction sequence and analyzed for fluorescence. No cell lysate proteins were found to be labeled, signifying no protein modification due to the copper exposure (Supporting Information Figure S8). However, some low-level fluorescence was detected with the wild-type GFP and other hexa-His-tagged proteins. We hypothesized this might be due to copper association with the hexa-His tag, leading to noncovalent association with the aminooxy fluorophore. This fluorescence was removed by washing with an EDTA solution, validating this hypothesis (see Supporting Information Figure S9). Fluorescence was still observed with samples possessing the diyne moiety after washing, providing further confirmation that the reaction occurs and is in fact site-specific labeling.

**Figure 2:**
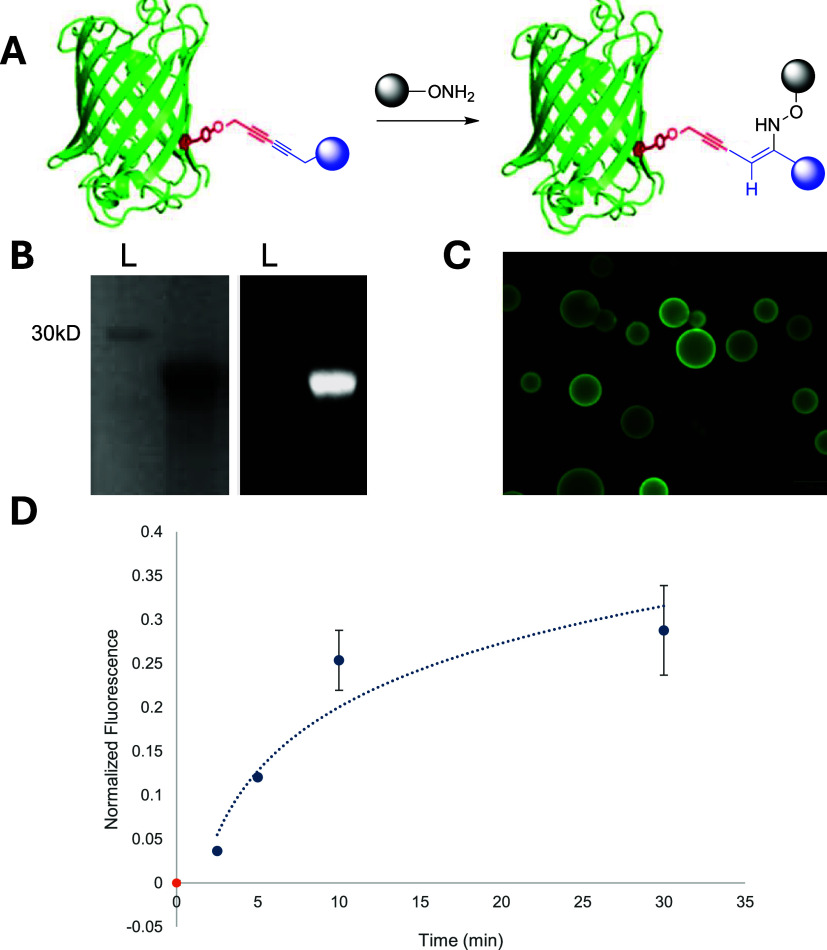
Trivalent conjugation using a Glaser–Hay produced conjugate with an aminooxy fluorophore. (A) GFP-*p*PrF-Biotin is reacted with an aminooxy fluorophore to produce a trivalent conjugate. (B) Denaturing SDS-PAGE analysis of the trivalent conjugate. Coomassie staining (left) indicates the presence of GFP. Fluorescence imaging of the gel (right) indicates successful fluorophore coupling as the GFP is denatured and no longer fluorescent. (C) Secondary confirmation of the successful reaction was determined using a streptavidin bead assay. Successful binding also demonstrated the successful coordination of both the biotin and the fluorophore, as the GFP was denatured prior to incubation with the bead. (D) Reaction timecourse determined by altering the duration of the reaction and then purifying the reaction using a Ni-NTA resin (see Supporting Information Figure S5). The different time points were analyzed by SDS-PAGE and densitometry measurements to ascertain the degree of coupling at each time point.^40^ Based on this experiment, complete trivalent formation occurs after approximately 30 min.

Given the successful preparation of a trivalent conjugate, we next aimed to optimize the reaction conditions. To investigate the reaction rate, we first performed a reaction timecourse at pH 6 over a 4 h period. Based on SDS-PAGE analysis, it appeared the reaction was approximately 85% complete within 5 min of incubation at 37 °C (see Supporting Information Figure S4). However, given that excess fluorophore was present as samples were heated to 95 °C to denature protein prior to electrophoresis, we redesigned the experiment. The GFP–biotin conjugate was first immobilized on a Ni-NTA resin, leveraging the hexa-His purification tag on the GFP, and then incubated with the aminooxy fluorophore for a given time period. The resin was then washed, and the protein conjugate was eluted and denatured to facilitate the SDS-PAGE analysis. This experiment demonstrated that the reaction is indeed rapid and reaches completion within 30 min at 37 °C (Figures 2D and S5). Conveniently, this conjugation also occurs in the absence of any metal catalyst, increasing its value as a practical bioorthogonal reaction. A screening of different pHs was also performed, and similar coupling efficiencies were observed between pH 6 to pH 8, suggesting that the reaction is tolerable within a wide pH range (see Supporting Information Figure S6). Ultimately, calculating protein concentration through densitometry/BCA assays and using fluorophore absorbance measurements to calculate fluorophore concentration via Beer’s Law, afforded the determination of conjugation yield to be 98% after 1 h. Moreover, mass spectrometry analysis of the protein demonstrated no mass of either the protein or the bivalent Glaser–Hay conjugate (see Supporting Information Figure S3), confirming a nearly quantitative conversion.

Control reactions with only the terminal alkyne-containing GFP-*p*PrF afforded no observable coupling, signifying the importance of the 1,3-diyne moiety. Moreover, the identity of the trivalent conjugate was confirmed by mass spectrometry (see Supporting Information Figure S3), indicating the addition of a single biotin and a single fluorophore to the conjugate. This confirmation also demonstrates that the coupling occurs due to the diyne functionality and not nonspecifically on the protein because of oxidations to ketones/aldehydes during any stage in the synthesis.

Due to the structural difference of the *O*-alkoxylamine from previously studied N-substituted hydroxylamines and the lack of a hydroxyl hydrogen to facilitate the Cope-type hydroamination, we next attempted to identify the chemical structure of our conjugate. Using a model small-molecule system of hexa-2,4-diyne-1,6-diol and *O*-methylhydroxylamine, initial attempts to isolate the product using various conditions and solvents were unsuccessful due to degradation during column chromatography. These challenges resulted in the transition to an NMR study in D_2_O to best mimic biological conditions and alleviate the need to isolate the products. These components were dissolved in D_2_O in a 1:1 molar ratio, and 1 equiv of NaOH was added to neutralize the commercially available *O*-methylhydroxylamine. Given the reaction’s stoichiometric nature and our inability to mimic the reagent excess that occurs in the protein conjugations, the reactions were heated to 65 °C for 12 h to drive the reaction to completion. Disappearance of starting material signals was observed with the appearance of new product resonances. Of note was the presence of a downfield signal at 8.26 ppm, suggesting the formation of a deshielded vinylic hydrogen and an enamine derivative (Supporting Information Figure S1). Consequently, we hypothesize that the reaction yields a single addition of *O*-alkyoxylamine to generate an alkynyl-enamine.

### One-Pot Cascade Reaction Optimization

After validation of the reaction, both on small-molecule substrates and on a protein, we explored the ability to conduct the multivalent cascade reactions in a single pot to further the bioconjugation’s utility. Several experimental variables were measured, including reaction time, reagent stoichiometric ratios, and reagent concentrations. Using the model GFP system, an initial Glaser–Hay coupling was performed with the biotin alkyne at room temperature in the presence of the CuI/TMEDA system. Reaction times ranged from 4 to 12 h, followed by the addition of the aminooxy fluorophore and further incubation. The reactions were then buffer exchanged into PBS using molecular weight cutoff spin columns and analyzed by SDS-PAGE and streptavidin binding assays. Excitingly, both reactions were found to be successful using the single-pot conditions (Figure 3). The standard Glaser–Hay incubation time of 4 h was required for diyne formation, and incubation of 15 min or more following aminooxy fluorophore resulted in the introduction of the fluorophore. The most important variable for optimal coupling was found to be the stoichiometric ratios of reaction partners, with a 3-equivalent excess of the aminooxy partner to the alkyne partner improving reaction yields. Moreover, doubling the concentrations of both partners resulted in greater than 90% production of multivalent conjugate as determined by SDS-PAGE. The heat denaturation of the trivalent conjugate to eliminate GFP fluorescence, followed by incubation with streptavidin resin, indicated the presence of both the fluorophore and the biotin partners in the one-pot process and corroborated fluorescence results observed from gel electrophoresis experiments (Figure 3).

**Figure 3:**
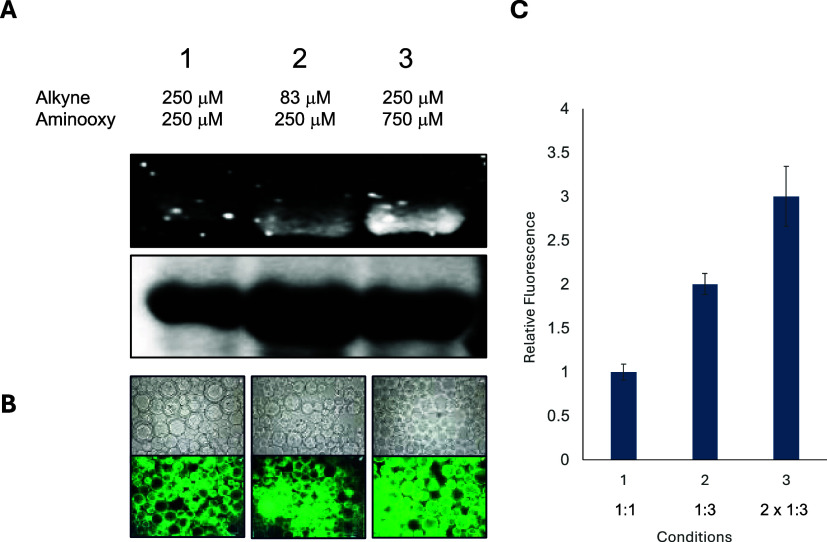
Optimization of a one-pot reaction to prepare a multivalent bioconjugate. The GFP-151-*p*PrF was reacted in the presence of both biotin alkyne and *O*-alkoxyamine fluorophore at various ratios in the presence of a Cu/TMEDA system. (A) Reaction progress was monitored by denaturing SDS-PAGE for the addition of the aminooxy fluorophore. Gel results indicated that a 1:3 ratio of alkyne partner to aminooxy partner afforded the highest degree of fluorescence. (B) Confirmation of biotin coupling was confirmed by the previously described streptavidin bead assay. (C) Densitometry measurements from the Coomassie Blue-stained gel image and the fluorescent image were used to assess the degree of coupling. All experiments identified that doubling partner concentrations in a 1:3 ratio represents the optimal conditions for one-pot coupling. Reactions were conducted in triplicate to provide the standard deviation.

### Application to Antibody–Drug Conjugates

Having developed a unique bioconjugation reaction to functionalize diyne-containing proteins with an aminooxy probe, we next sought to demonstrate the utility of the approach in a practical application. For these experiments, the fragment antigen-binding (Fab) region of the monoclonal antibody trastuzumab (anti-HER2-Fab) was selected due to its demonstrated clinical success in the treatment of HER2-positive breast cancer. Specifically, serine residue 202 was selected for the incorporation of an ncAA based on the residue’s surface exposure and precedence for ncAA incorporation.^64,65^ Initially, we prepared a methotrexate (MTX) alkyne derivative through amide coupling of the two carboxylic functionalities with propargyl amine. The U.S. Food and Drug Administration has approved MTX as a dihydrofolate reductase inhibitor toward the treatment of cancer.^66^ Combining the potency of these two therapeutics results in a conjugate poised to have powerful anticancer activity, and introducing a third partner would further expand its therapeutic potential. For initial proof-of-concept experiments, the third partner was selected to be the AlexaFluor-488 aminooxy fluorophore to track therapeutic delivery.

The anti-HER2-Fab was first expressed with *p*PrF at residue 202 in accordance with literature precedent of the preparation of bivalent conjugates.^67^ Successful expression of the *p*PrF-containing Fab was confirmed by SDS-PAGE (Figure 4). Glaser–Hay coupling of the anti-HER2-*p*PrF-Fab was then performed with the MTX-alkyne, followed by sequential coupling with the AlexaFluor-488 aminooxy fluorophore. After purification and confirmation of the successful preparation of the trivalent conjugate (Supporting Information Figure S10), viability screens of the MTX-fluor-Fab in HER2+ human breast cancer cell lines BT-474 failed to decrease the viability (Supporting Information Figure S12). This failure was hypothesized to be either due to the inability to reach MTX’s cytotoxic concentrations within the cell or due to the alkynyl modifications decreasing the potency of the drug.

**Figure 4:**
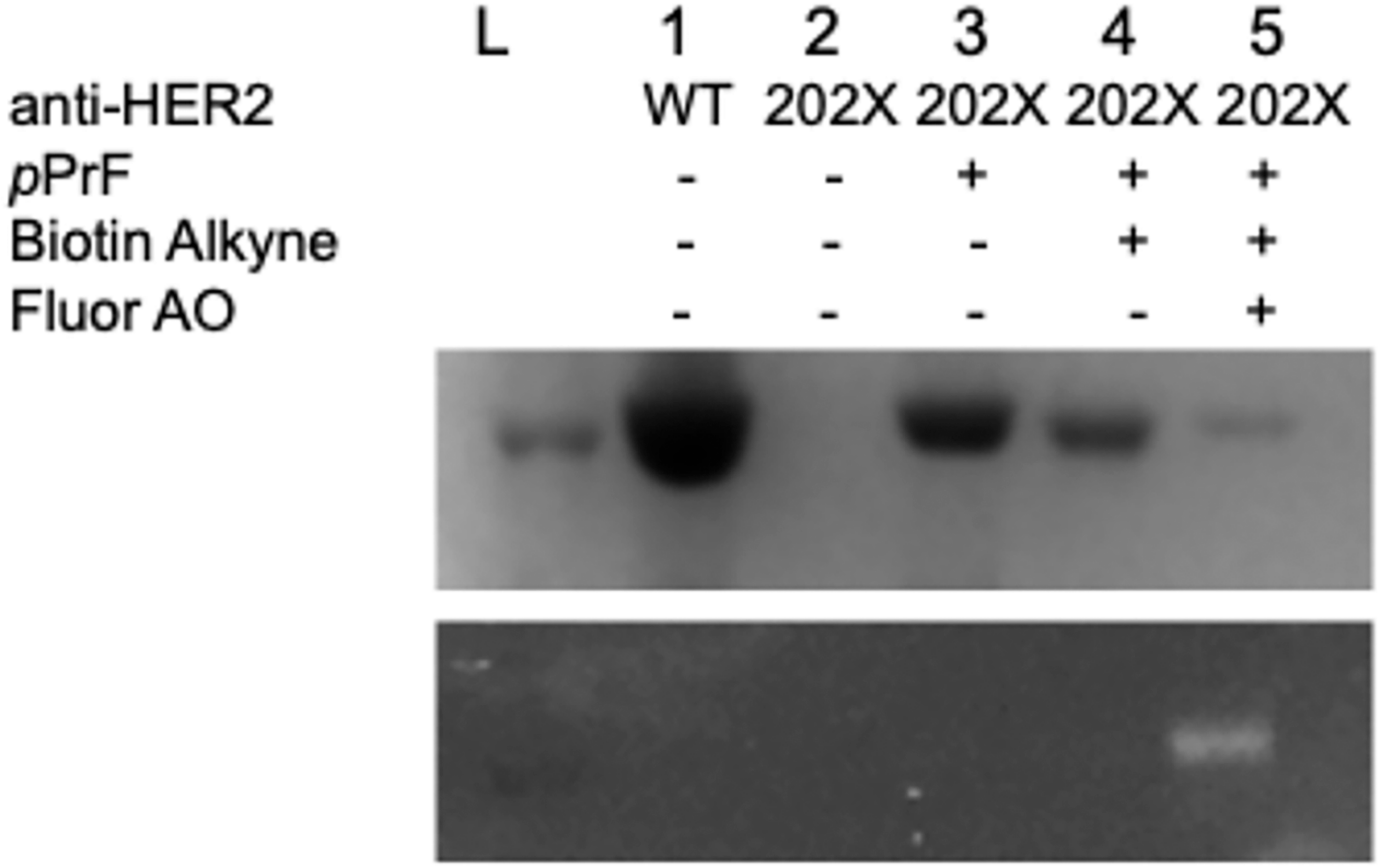
Preparation of the anti-HER2-Fab multivalent conjugates. Denaturing SDS-PAGE confirmation of anti-HER2 expression and conjugation. Wild-type anti-HER2 (lane 1) serves as a positive control of protein expression. In the expression containing the anti-HER2 with a TAG mutation at residue 202, the absence of the ncAA *p*PrF results in no protein expression (lane 2); however, protein is observed if *p*PrF is added to the expression culture (lane 3). Reaction with biotin alkyne under Glaser–Hay conditions still results in the observation of the protein (lane 4). Final reaction with *O*-alkoxyamine fluorophore produces a fluorescently labeled protein signifying coupling (lane 5; bottom gel). Fluor AO = *O*-alkoxyamine fluorophore.

Consequently, we opted to alter the cytotoxic payload of the conjugate to the more potent monomethyl auristatin E (MMAE). Auristatins have more commonly been employed in immunotherapeutics due to their picomolar potency in disrupting microtubule formation. A commercially available acetylene-linker-Val-Cit-PABC-MMAE was obtained and used with the previously established protocol to prepare a trivalent conjugate with the aminooxy derivatized fluorophore. Although SDS-PAGE could not be used to verify the synthesis of the anti-HER2-Fab-MMAE conjugate, successful coupling of the aminooxy fluorophore was confirmed by SDS-PAGE and fluorescence imaging (Supporting Information Figure S13). The presence of the fluorescence is only possible if a diyne functionality is installed, confirming the success of both the Glaser–Hay coupling and the aminooxy conjugation (Figure 4). The preparation of the trivalent conjugate was also confirmed by mass spectrometry.

To confirm the functionality of the trivalent conjugate, *in vitro* assays were performed via incubation of the conjugate with the HER2 protein. The anti-HER2-Fab-MMAE-fluorophore conjugate and unreacted anti-HER2-Fab-*p*PrF protein were separately incubated with human HER2 and then analyzed by nondenaturing SDS-PAGE (Figure 5A). Fluorescence imaging coupled with Coomassie staining indicates that a fluorescence band was present when the trivalent conjugate was incubated with HER2, demonstrating recognition and binding of the Fab with the HER2 protein. With confirmation of its functionality, the conjugate’s effect on cell viability was then assessed. Gratifyingly, the therapeutic was able to specifically target the BT-474 (HER2+) cells while not decreasing the viability of HeLa (HER2- cervical cancer) cells (Figures 5 and S14). Using a Cytotox NIR reagent (Inucyte) to identify cell viability, the *p*PrF-containing Fab alone had no effect on cellular viability over time and only minimal effect at high concentrations. Conversely, both the divalent anti-HER2-MMAE and the trivalent anti-HER2-MMAE-fluor resulted in a marked decrease in cellular viability (Figure 5C). Using Desmos to fit the data, EC_50_ values of 3.44 nM (divalent) and 2.72 nM (trivalent) were calculated; however, the difference between the two is within the standard error. Interestingly, slight variability was observed between the two MMAE conjugates, with the trivalent fluorophore conjugate being slightly less efficacious. This difference is likely due to the potential decreased activity of the MMAE or the addition of the fluorophore, causing steric interference. Full effect of the conjugate was observed after 10 h of incubation with BT-474 cells, and both MMAE conjugates had nanomolar potency, as observed with a dose–response curve (Figure 5D). The targeting of the trivalent conjugate was also visualized by fluorescence microscopy, as both HeLa and BT-474 cells were incubated with the anti-HER2-MMAE-fluor conjugate and monitored by fluorescence microscopy over a 12 h period (Figure 5). Within 15 min, fluorescence localization was observed on the surface of the HER2+ BT-474 cells. Moreover, internalization of fluorescence appeared to occur after approximately 2 h, and most cells appeared nonviable after 8 h. Comparable fluorescence localization and decreased viability were not observed with the HER2-HeLa cells (Figure 5B).

**Figure 5:**
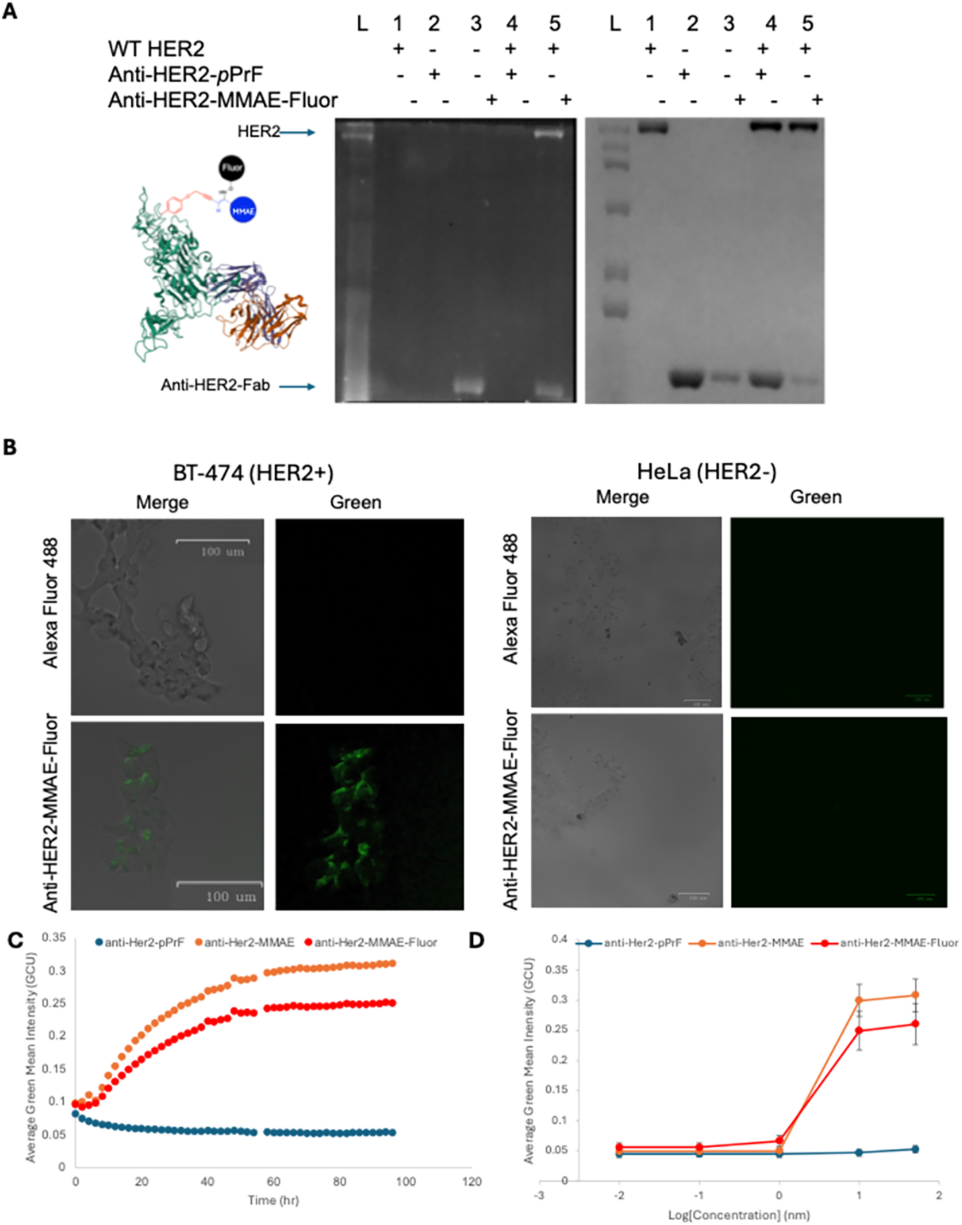
Development and assessment of a trivalent antibody–drug conjugate. (A) Construction and binding of a trivalent anti-HER2-Fab-MMAE-fluorophore and assessment by native SDS-PAGE. The HER2 receptor was purchased (VWR) and used as a control to assess molecular weight (lane 1). Initially, the anti-HER2-*p*PrF was expressed (lane 2) and reacted with both the MMAE-alkyne and the *O*-alkoxylamine fluorophore to yield the trivalent conjugate (lane 3), resulting in a fluorescent anti-HER2-Fab (left). To visualize HER2/anti-HER2-Fab binding, the ncAA-containing Fab (lane 4) and the trivalent Fab (lane 5) were incubated with HER2. The decreased band intensity of the Fab and the production of a fluorescent band at the top of the gel indicated that the trivalent Fab was functional and able to associate with its target protein. (B) Functional HER2 binding with fluorescent trivalent MMAE-Fab. HER2+ BT-474 (left) or HER2- HeLa (right) cells were incubated with either *O*-alkoxylamine fluorophore (top) or the anti-HER2-Fab-MMAE-fluorophore conjugate for 10 min and imaged for fluorescence. Cell surface localization of the fluorescent signal was only detected in the case of the trivalent conjugate and is not a result of nonspecific fluorophore interactions. No labeling was observed with the HeLa cells. (C) Viability assay of BT-474 cells with Cytotox NIR reagent. Lethality was detected for both the bivalent anti-HER2-MMAE construct (orange) and the trivalent anti-HER2-MMAE construct (red) after approximately 60 h, but no decreased viability was detected for the anti-HER2 construct (blue) harboring no drug. No decreases in viability were observed over similar time points in HER2-negative cells (Supporting Information Figure S14). (D) Dose–response curves for the three constructs with BT-474 cells demonstrating nanomolar EC_50_ efficacy values for the bivalent and trivalent constructs.

## Conclusion

Overall, we have identified a new bioconjugation reaction to expand the chemical toolbox and generate multivalent conjugates. Importantly, the alkyne and aminooxy conjugation partners are plentiful and commercially available due to their utilization in previously developed conjugation reactions. The linking of an aminooxy group with a 1,3-diyne progresses rapidly, forms a stable covalent linkage, and requires no additional reagents. As a result of all of these factors, this approach should have widespread applicability to the preparation of therapeutic and diagnostic bioconjugates. Moreover, employing ncAAs in this reaction facilitates the introduction of multiple new functionalities to proteins in a highly specific and homogeneous fashion using a single ncAA. These results represent an advancement in the field, and the utility of these conjugates has been demonstrated through the preparation of a trivalent immunoconjugate that retains cytotoxic activity and whose drug delivery is trackable. Due to specificity and rapid reaction rate, this cascade approach to multivalent conjugates harbors significant potential, and future work aims to expand the range of reaction partners and broaden the applications of the conjugates.

## Supplementary Material



## Notes

Experimental protocols, MS data, ubiquitin–streptavidin bead assays, timecourse SDS-PAGE gels, pH screening experiments, full SDS-PAGE gels of main manuscript figures, control experimental results, and additional Fab expression and conjugation validation SDS-PAGE gels. (PDF)

## Abbreviations

ncAA: noncanonical amino acid
GFP: green fluorescent protein
Ub: ubiquitin
PBS: phosphate buffered saline
HER: Human Epithelial Growth Factor Receptor
*p*PrF: para-propargyloxyphenylalanine
AO: aminooxy
MMAE: monomethyl auristatin E
