# Supplementary material for: Development and Optimization of an Aminooxy Coupling Reaction to Prepare Multivalent Bioconjugates with a Single Noncanonical Amino Acid
Source: Bioconjug Chem. 2026 Jan 20;37(2):271–80. doi: 10.1021/acs.bioconjchem.5c00517 (PMC12921668; doi:10.1021/acs.bioconjchem.5c00517)
Supplement: Supplementary file 1 [file bc5c00517_si_001.pdf]

Supporting Information for

Development and Optimization of an Aminooxy Coupling Reaction to Prepare Multivalent Bioconjugates with a Single Noncanonical Amino Acid

Robert K. Gourdie ‡,^, Emily L. Boyt ‡,^, Brian M. Flood^, Alexander C. Willard^, William I. Eisen^, Tyler L. Skeen^, Annalee R. Hassler^, Aaron S. Wang^, Cedrick R. Dimaranan^, Sophia K. Rothman^, Elizabeth A. King∫, Jonathan C. Maza˥, and Douglas D. Young*,^

^Department of Chemistry, William & Mary, Williamsburg, Virginia 23185 USA
∫ Department of Chemistry, University of California Berkeley, Berkeley, California 94720 USA
˥ Department of Chemistry, University of California San Francisco, San Francisco, California 94143 USA

* Corresponding author

‡Authors contributed equally to the work

Table of Contents:

Experimental Section……………………………………………………………..1
Supporting Figures………………………………………………………………..8

Experimental

General. Solvents and reagents, including the AlexaFluor-488-Aminooxy, acetylene-linker-Val-Cit-PABC-MMAE, and biotin-alkyne, were obtained from either Sigma Aldrich, Fisher Scientific, or VWR and used without further purification. Cancer cell lines were obtained from ATTC. Streptavidin resin was obtained from G-Biosciences. Plasmids were obtained from the laboratory of Dr. Peter Schultz at The Scripps Research Institute. Reactions were conducted under ambient atmosphere with solvents directly from the manufacturer. All GFP proteins were purified according to manufacturer's protocols using a Qiagen Ni-NTA Quik Spin Kit. SDS-PAGE was performed using a BioRad mini-PROTEAN Tetra system and visualized on a BioRad gel imaging system. Resins were analyzed for fluorescence using a BioRad ZOE fluorescent inverted microscope. Samples were analyzed on an Agilent 6520 Accurate-Mass Quadrupole-Time-of-Flight (Q-TOF) mass spectrometer equipped with an electrospray (ESI) ionization source and liquid chromatography (LC) (Agilent). Ionization settings were: positive mode; capillary voltage 3500 kV; fragmentor voltage 200 V; drying gas temperature 350 °C. Additional conjugates were anaylzed on a Voyager time of flight (TOF) mass spectrometer equipped with a matrix-assisted laser desorption/ionization (MALDI) ionization source. No unexpected or unusually high safety hazards were encountered in any of the reported experiments.

Synthesis of p-propargyloxyphenylalanine (pPrF).

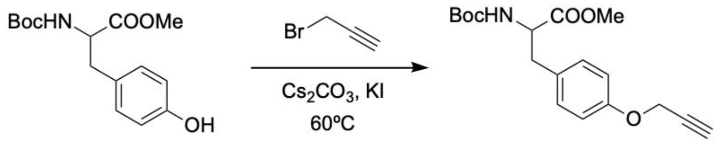

Boc-Tyrosine-OMe (0.500 g, 1.69 mmol) and cesium carbonate (0.826 g, 2.54 mmol) were added into a flame-dried vial with a magnetic stir bar and dissolved in dry DMF (10 mL). This mixture was then stirred at 60ºC for 30 minutes. Propargyl bromide (0.452 mL, 5.10 mmol) and a spatula tip of KI were then added to the vial. The reaction was allowed to stir at 60ºC overnight and then cooled to room temperature and filtered. Next, the filtered reaction mixture was diluted with brine (20 mL) and extracted with dichloromethane (3 x 20 mL). The organic layer was washed with brine (3 x 20 mL), dried over $MgSO_4$, and concentrated *in vacuo*. The resulting oil was purified via flash chromatography (25% EtOAc in hexanes) and concentrated to yield protected *p*-propargyloxyphenylalanine (*p*PrF) as a white crystal (0.464 g, 83% yield). ^{1}H NMR (400 MHz, $CDCl_3$): δ 7.02 (d, 2 H), 6.82 (d, 2 H), 4.95 (d, 1 H), 4.53 (d, 2 H), 4.27 (d, 1 H), 3.71 (s, 3 H), 3.02 (m, 2 H), 2.39 (t, 1 H), 1.41 (s, 9 H). ^{13}C NMR (400 MHz, $CDCl_3$): δ 172.4, 157.9, 130.3, 127.9, 114.5, 83.5, 79.9, 68.8, 66.0, 54.5, 52.2, 37.4, 28.3, 28.2, 21.1, 15.1

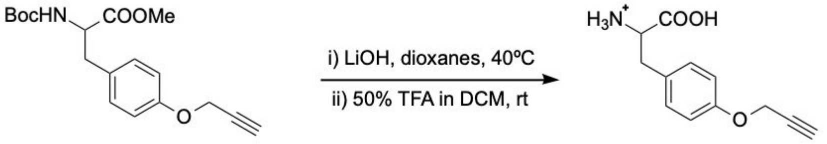

To remove the methyl protecting group, 3 mL of a 1:1 solution of 1M LiOH in water and dioxanes was added to protected *p*PrF (0.464 g., 0.73 mmol) in a vial on ice. The mixture was then stirred at room temperature for two hours and the dioxanes were removed *in vacuo*. The aqueous solution was cooled on ice and 6 M HCl was added dropwise until a pH of 4 was achieved and a solid white precipitate formed. The mixture was then extracted with cold ethyl acetate and the organic layer was washed with cold water. The organic layer was then dried over $MgSO_4$ and concentrated under reduced pressure to afford a yellow oil. This yellow oil was then dissolved in a 50% trifluoroacetic acid in dichloromethane on ice and allowed to stir at room temperature for one hour. The solvent was removed *in vacuo,* affording the trifluoroacetate salt of *p*PrF as a brown solid (0.301 g., 98% yield). ^{1}H NMR (400 MHz, CD_3OD): δ 7.19 (d, 2 H), 6.93 (d, 2 H), 4.91 (s, 1 H), 4.14 (m, 1 H), 3.02 (m, 2 H), 2.54 (t, 1 H). ^{13}C NMR (400 MHz, $CDCl_3$): δ 168.4, 155.7, 128.6, 125.4, 113.5, 73.4, 54.4, 52.3, 45.4, 25.5.

General Expression of pPrF-Containing Proteins. Using an Eppendorf electroporator set to 1800 V, *Escherichia coli* BL21(DE3) cells were co-transformed with a pET-GFP-TAG-151 or pET-UB-TAG-48 plasmid (0.5 µL) and pEvol-*p*CNF plasmid (0.5 µL) and allowed to recover for one hour in LB Media at 37 ºC. Following recovery, cells were plated on an LB-Agar plate supplemented with ampicillin (50 mg/mL) and chloramphenicol (34 mg/mL) and grown at 37°C for 16 hours. Next, a single colony from the plate was used to inoculate 5 mL LB media also supplemented with ampicillin and chloramphenicol. The culture was grown to confluence at 37°C for 16 hours. The dense culture was used to begin an expression culture in LB Media (25 mL) supplemented with ampicillin and chloramphenicol at an OD_{600} of 0.1. The expression culture was shaken at 37 ºC until reaching an OD_{600} of 0.7-0.9. Upon reaching this density, protein expression was induced by the addition of IPTG (1M, 25 µL), arabinose (20%, 25 µL) and pPrF (100mM, 250 µL). The cells were then left to shake at 37 ºC for 16 hours and then were pelleted by centrifugation (10 min, 5000 rpm), after which the supernatant was discarded and the pellet was stored at -80º C for at least 20 minutes. The GFP was then purified using a Qiagen Ni-NTA Quik Spin Kit according to the manufacturer's protocol. Protein yield and purity were assessed via SDS-PAGE and spectrophotometrically via a Nanodrop spectrophotometer. Finally, the mutant proteins were buffer exchanged into phosphate buffered saline (PBS;10 mM Na_2HPO_4, 2 mM KH_2PO_4, 2.7 mM KCl, 137 mM NaCl, pH = 7.0) using concentration columns (10000 MWCO for GFP mutants, 5000 for ubiquitin mutants).

*Expression of anti-HER2-Fab-LC202-*p*PrF.* Using an Eppendorf electroporator set to 1800 V, *Escherichia coli* BL21(DE3) cells were co-transformed with a pBad-antiHER2Fab-LC202TAG plasmid (0.5 µL) and pEVOL-*p*CNF plasmid (0.5 µL) and allowed to recover for one hour in LB Media at 37ºC. Following recovery, cells were plated on an LB-Agar plate supplemented with ampicillin (50 mg/mL) and chloramphenicol (34 mg/mL) and grown at 37°C for 16 hours. Next, a single colony from the plate was used to inoculate 5 mL 2XYT media also supplemented with ampicillin and chloramphenicol. The culture was grown to confluence at 37°C for 16 hours. The dense culture was used to begin an expression culture in 2XYT Media (25 mL) supplemented with ampicillin and chloramphenicol at an OD_{600} of 0.1. The expression culture was shaken at 37ºC until reaching an OD_{600} of 0.7-0.9. Upon reaching this density, protein expression was induced by the addition of IPTG (1M, 25 µL), arabinose (20%, 25 µL) and *p*PrF (100mM, 250 µL). The cells were then left to incubate at room temperature for 16 hours and then were pelleted by centrifugation (10 min, 5000 rpm), after which the supernatant was discarded and the pellet was stored at -80ºC for at least 20 minutes. The cells were resuspended and lysed in sodium phosphate buffer (0.387 M Na_2HPO_4, 0.113 M $NaHPO_4$, 0.15 M NaCl, pH 7.4) via sonicating for 4 x 10 s on ice and then pelleted by centrifugation for 10 minutes at 5000 rpm. Protein G resin (1 mL; G Biosciences) was loaded into a protein purification column and equilibrated with sodium phosphate buffer. Following centrifugation, all of the cell lysate supernatant was passed over the resin at least two times to allow the anti-Her2-Fab-LC202*p*PrF to bind to the resin. The resin was then washed with sodium phosphate buffer (3 x 1 mL), centrifuging for 1 minute at 1800 rcf each time. Finally, the anti-Her2-Fab-LC202*p*PrF was eluted by pipetting 65 µL of Tris-HCl buffer (1 M, pH 9) into the collection tube and 500 µL of glycine-HCl buffer (0.2 M, pH 2.5) onto the resin. The protein purification column was immediately spun for 1 minute at 1800 rcf to allow the glycine-HCl eluent to flow into the collection tube and be neutralized by the Tris-HCl buffer. This elution step was repeated once more. Protein yield and purity were assessed via SDS-PAGE and spectrophotometrically via a Nanodrop spectrophotometer. Finally, the protein was buffer exchanged into PBS (10 mM Na_2HPO_4, 2 mM KH_2PO_4, 2.7 mM KCl, 137 mM NaCl, pH 6) using concentration columns (10k MWCO).

General Glaser-Hay Bioconjugation of pPrF-containing proteins. CuI (7.2 mg, 0.038 mmol) and TMEDA (76 µL, 0.455 mmol) were combined in DI water (302 µL). This mixture was then sonicated of CuI and heated at 60ºC for 15 minutes. After heating, the mixture was vortexed and cooled on ice. Next, *p*PrF mutant protein (25 µL, ~1 mg/mL, PBS pH 6) and catalase (7.5 µL, 9 mg/mL in PBS pH 6) were added. The CuI/TMEDA mixture was vortexed again to resuspend any solid CuI, and 5 µL of the CuI/TMEDA mixture were added to the PCR tube. The PCR tube was then heated at 37ºC for 15 minutes. Alkyne probe (12.5 µL, 1 mM; i.e. biotin alkyne, MTX-alkyne, or acetylene-linker-Val-Cit-PABC-MMAE (MMAE-alkyne)) was added next to the PCR tube. The tube was sealed and the reaction was shaken at room temperature for 4 hours. Finally, the reaction was added to a concentrator column (10k MWCO, Corning Spin-X) that was hydrated with PBS (10 mM Na_2HPO_4, 2 mM KH_2PO_4, 2.7 mM KCl, 137 mM NaCl, pH 6). The reaction washed with additional PBS (8 x 200 µL) and concentrated to a final volume of 25 µL. Protein conjugate was analyzed by streptavidin bead binding assay and/or SDS-PAGE to assess the bioconjugation.

Optimized Addition of Aminooxy Probes to Glaser-Hay Conjugates. Glaser-Hay conjugate (25 µL, ~1 mg/mL, PBS pH 6) and aminooxy probe (12.5 µL, 1 mM; i.e. Alexa Fluor 488 hydroxylamine) were combined in a PCR tube and shaken for 30 minutes at 37ºC. The reaction was added to a concentrator column (10k MWCO, Corning Spin-X) that was hydrated with PBS (10 mM Na_2HPO_4, 2 mM KH_2PO_4, 2.7 mM KCl, 137 mM NaCl, pH 6). The reaction mixture was washed with additional PBS (8 x 200 µL), then concentrated to a final volume of 25 µL. Protein conjugate was analyzed by SDS-PAGE and MS to assess the bioconjugation.

Timecourse protocol. GFP Glaser-Hay conjugate (25 µL, ~1 mg/mL, PBS pH 6) and aminooxy probe (12.5 µL, 1 mM; i.e. Alexa Fluor 488 hydroxylamine) were combined in a PCR tube and shaken for 5, 15, 30, 45, 60, 90, 120, 180, 240, or 360 minutes at 37ºC. During incubation Ni-NTA resin (200 µL) was equilibrated with PBS pH 6, and reactions were quenched by addition to the Ni-NTA column with a 10 min incubation at room temperature. The resin was washed 3 times according to manufacturer protocols and then proteins were eluted with imidazole containing buffer. Protein conjugate was then analyzed by SDS-PAGE and fluorescence was measured using a BioRad imager. The gels were then stained with Coomassie Blue and

re-imaged to use densitometry measurements of stain vs. fluorescence to quantify degree of fluorophore conjuation.

Biotin Binding Assays. In order to ascertain the success of the biological bioconjugation reactions, immobilized Streptavidin resin (G-Biosciences; 30 μL) was added to a PCR tube and pelleted in a tabletop centrifuge (Eppendorf). The supernatant was removed, and the resin was equilibrated by resuspension in 50 μL of PBS and pelleted again. This equilibration was repeated twice, removing the supernatant after each time. Next, conjugation product (5 μL) was added directly into the resin pellet. The tube was then sealed and incubated at room temperature for 30 minutes. The resin was then pelleted and washed 5 times with PBS (200 μL) in the same manner as above to remove any unbound protein. The beads were then imaged with a BIO-RAD ZOE™ fluorescent cell imager to quantitate bead fluorescence due to the presence of the biotin and the fluorescent probe.

Characterization of the Reaction Between 1,3-Diynes and Aminooxy Functionalities. hexa-2,4-diyne-1,6-diol (22.4 mg, 0.20 mmol) was added to a vial and dissolved in as little D_2O as possible. O-methylhydroxylamine hydrochloride (16.7 mg, 0.20 mmol) and NaOH (8 mg, 0.20 mmol) were then added to this solution. The reaction was allowed to stir at 65ºC for 16 hours and then analyzed via ^{1}H NMR (400 MHz; Figure S1). Additionally, headspace vapor from the reaction was sampled and analyzed by direct injection into a GC/MS, resulting in the identification of the expected mass at 157.2 corresponding to the proposed structure (Figure S2).

A

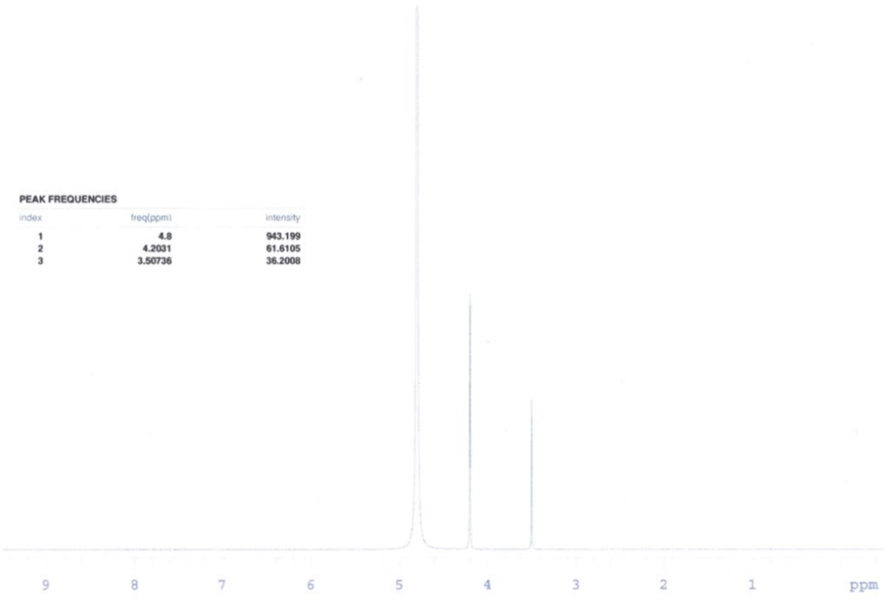

B

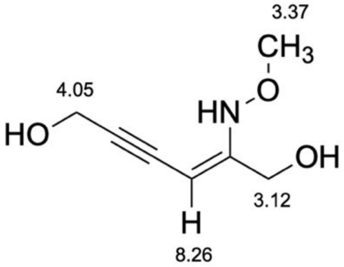

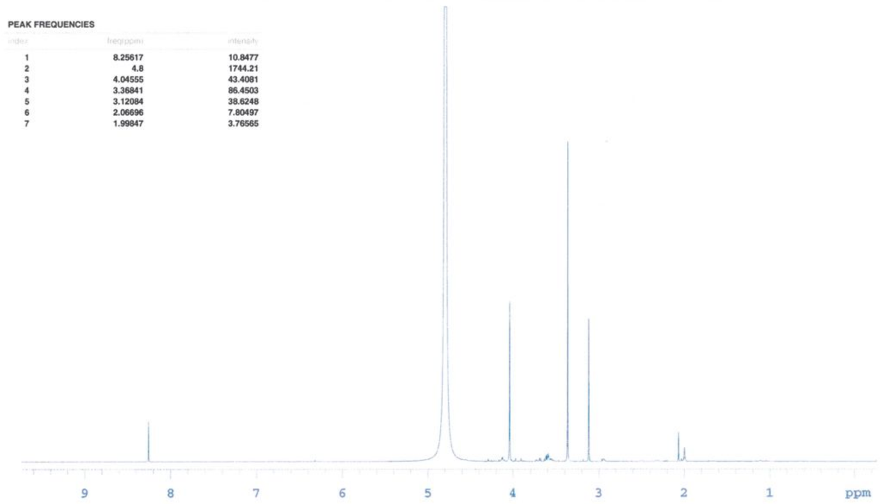

Figure S1. ^{1}H NMR spectra of hexa-2,4-diyne-1,6-diol and O-methylhydroxylamine hydrochloride combined in D_2O in the presence of NaOH. The signal at 4.20 ppm corresponds to the methylene protons on the diyne while the signal at 3.51 ppm corresponds to the methyl protons of O-methylhydroxylamine. Chemical shifts were normalized to the H_2O signal (4.8 ppm). **B.** ^{1}H NMR spectra of the reaction between hexa-2,4-diyne-1,6-diol and O-methylhydroxylamine hydrochloride in the presence of NaOH. The presence of new signals at 8.26, 4.05, 3.37, and 3.12 ppm were noted. Chemical shifts (in ppm) from the ^{1}H NMR spectra in Figure 3.12B have been assigned to their believed corresponding protons.

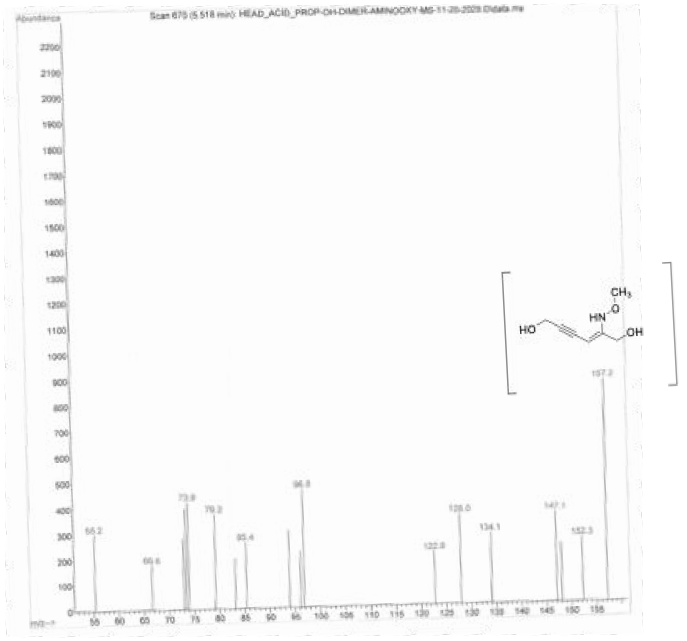

Figure S2. GC/MS analysis of headspace vapor of the reaction indicated the expected mass of the proposed product at 157.2 amu. Additional propargyl fragments are also observed at 55.2 amu.

Synthesis of MTX-Alkyne

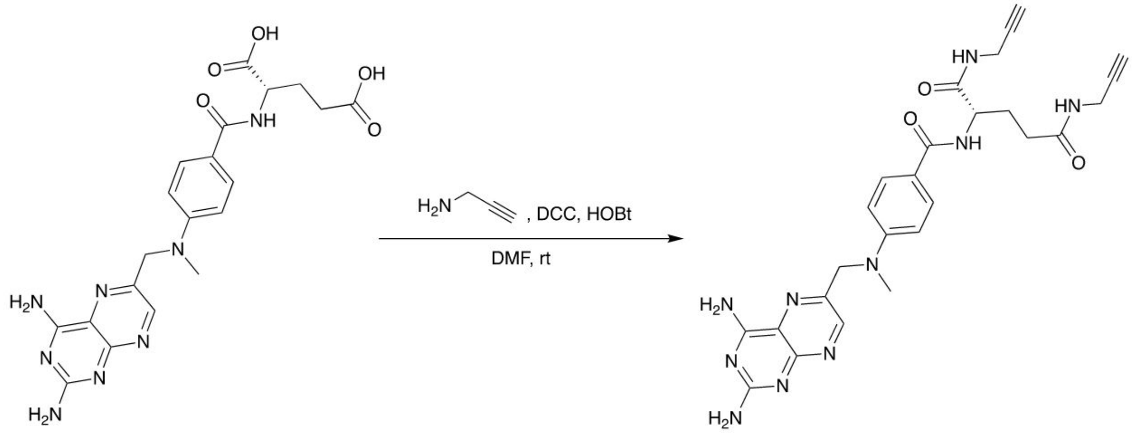

Methotrexate (90 mg, 0.2 mmol) was added to a vial with a magnetic stir bar and dissolved in 3 mL DMF. The solution was cooled to 0ºC, and triethylamine (63 µL, 0.45 mmol) was added to the vial. Next N,N'-dicyclohexylcarbodiimide (DCC) (0.103 g, 0.50 mmol) was added, and the reaction mixture was stirred on ice for 15 minutes. Following this period, propargylamine (27 µL, 0.42 mmol) was added to the vial. The reaction was stirred on ice for another 2 hours and then allowed to come to room temperature overnight. The reaction mixture was diluted with water so that a yellow solid precipitated out of solution. The mixture was vacuum filtered to isolate and dry the yellow solids. After drying, the yellow solids were dissolved in methanol, which was dried with sodium sulfate and filtered. The solution was analyzed by thin layer chromatography (15% methanol in dichloromethane) to determine reaction success. The reaction was purified by dry-loaded flash chromatography (15% methanol in dichloromethane) to afford methotrexate-bis-(propargylamide) (MTX-alkyne) as a bright yellow solid (44 mg; 42%). ^{1}H NMR (400 MHz, CD_3OD) δ 8.56 (s, 1H), 7.77 (d, 2H), 6.87 (s, 1H), 4.85 (s, 2H), 4.49 (m, 1H), 3.97 (s, 2H), 3.90 (s, 2H), 3.26 (s, 3H), 2.57 (s, 1H), 2.53 (s, 1H), 2.34 (m, 1H), 1.24-1.18 (m, 2H), 2.19-2.13 (m, 2H), 1.17-1.05 (m, 1H).

*Incucyte-based antibody-drug conjugate cell killing assay***.** The HER2+ breast-cancer cell line BT474 was plated out at 10,000 cells/well in a clear-bottom 96-well TC plate suitable for cell imaging in complete DMEM. Cells were allowed to adhere overnight at 37 ºC with 5% CO_2. The next day, media was removed and cells were resuspended in 100 µL of complete DMEM supplanted with the appropriate experimental conditions and previously filtered using a 0.2 µm syringe tip filter (Biotium). Briefly, these conditions were the Her2 Fab-pPrF, Her2 Fab-pPrF with conjugated auristatin, and Her2 Fab-pPrF with conjugated auristatin followed by conjugated AlexaFluor-488 at concentrations of either 50, 10, 1, 0.1, 0.01 nM. A working solution of Cytotox NIR dye (Incucyte) was prepared at 3 µM and 1 µL was added to each well for a final concentration of 30 nM. Samples were placed in an IncuCyte live cell imager (Sartorius) at 37 ºC with 5% CO_2. Images were taken every 2 hrs using the phase, green, and NIR channels. Analysis of NIR signal (cell death) was performed using the IncuCyte Analysis software.

HeLa MTT cell proliferation assay. Human HeLa cell line was obtained from the ATCC (ATCC, Manassas, USA). HeLa cells were maintained in Dulbecco's Modified Eagle's Medium (DMEM) (Gibco, Grand Island, New York, United States) supplemented with 10% by volume fetal bovine serum (Gibco, Grand Island, New York, United States) and 1% by volume penicillin/streptomycin. The cells were incubated in a 5% CO_2 incubator (New Brusnwick Galaxy 48 R, Eppendorf, Edison, New Jersey) at 37 °C.

The cell viability of the HER2-Auristatin-Aminoxyfluor was measured using a MTT Cell Proliferation kit (Oz Biosciences, Marseille, France). HeLa cells were seeded in a 96-well plate with 200 µL of supplemented DMEM and incubated for 24 hours. After incubation the DMEM was replaced, and wells were treated in triplicate with experimental conditions at a final concentration of 1ng/mL. (trivalent in PBS 6, a positive control of Auristatin dissolved in DMSO 1 ng/mL, and a negative control of DMSO). The cells were incubated with the test compound and controls for another 24-hour period. After incubation the DMEM was removed and 100 µL of 1x MTT working solution was added. The plate was incubated at 37 °C for 4 h. After the incubation period 100 µL of solubilization solution was added. The plate sat for an additional 15 minutes and then the absorbance was measured at a wavelength of 570 nm and a reference wavelength at 650 nm

using the SpectraMax iD3 (Molecular Devices, San Jose, CA, USA). The data were exported and analyzed in Microsoft Excel™ .

BT-474 Fluorescence microscopy assay. The BT-474 cell line was obtained from the ATCC (ATCC, Manassas, USA). The BT-474 cells were maintained in 46-X Hybri-Care medium (Hybri-Care) (ATCC, Manassas, USA) supplemented with 10% by volume fetal bovine serum (Gibco, Grand Island, New York, United States) and 1.5 g of sodium bicarbonate. The cells were incubated in a 5% CO_2 incubator (New Brusnwick Galaxy 48 R, Eppendorf, Edison, New Jersey) at 37 °C.

BT-474 cells were seeded in a 96-well plate with 200 µL of supplemented Hybri-Care media and incubated for 48 hours. After incubation the Hybri-Care media was replaced and several wells were treated the HER2-Auristatin-Aminoxyfluor trivalent in PBS 6 was added (final concentration 1 ng/mL). Treated wells were monitored every 30 minutes over the course of six hours and imaged wells were washed with d-PBS three times 200 uL and monitored for fluorescence. Fluorescence imaging of the BT-474 cells was imaged with the ZOE™ Fluorescent Imager (BIORAD, Berkley, USA).

Figures

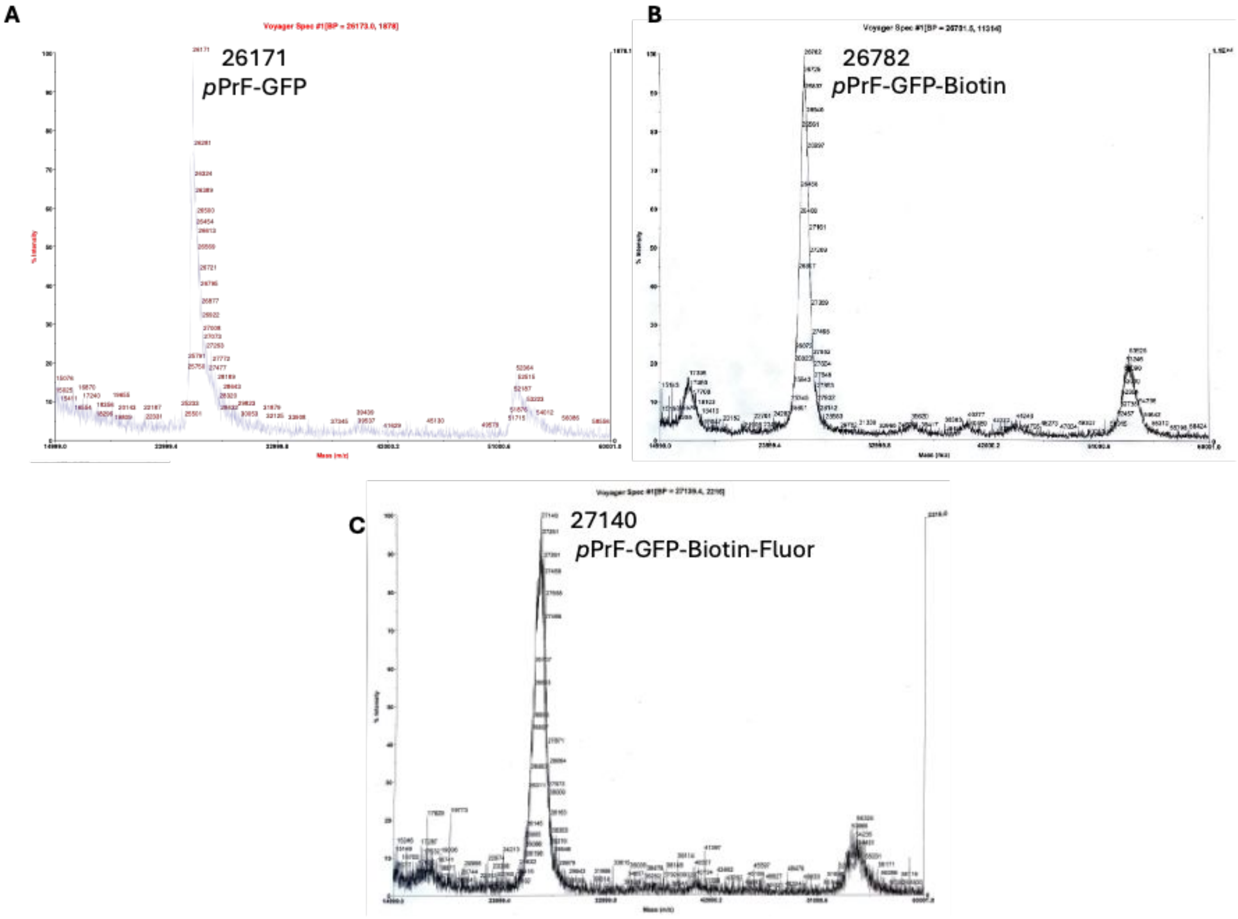

Figure S3. Mass spectrometry analysis of proteins. A. GFP containing the pPrF ncAA as a baseline mass. B. Divalent conjugate of pPrF-GFP with biotin alkyne via a Glaser-Hay bioconjugation. C. A correct increase in the m/z ratio of sfGFP-151-*p*PrF (m/z = 26167) indicated the successful synthesis of a multivalent conjugate (observed m/z = 27139.4; expected m/z=27138) that contained a single biotin alkyne moiety and a single Alexa Fluor 488 hydroxylamine moiety.

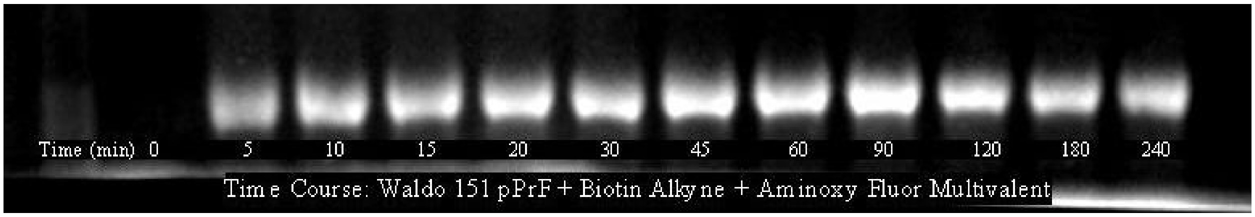

Figure S4. Preliminary experiments revealed that the reaction of the GFP-biotin conjugate and Alexa Fluor 488 hydroxylamine occurred in a robust fashion, prompting additional experimentation to more accurately gauge the reaction speed and to mitigate potential interference from excess fluorophore. Additionally, some concern that lack of removal of unreacted fluorophore may be driven to completion during the denaturation stage of SDS-PAGE prompted further examination of the timecourse (Figure S5).

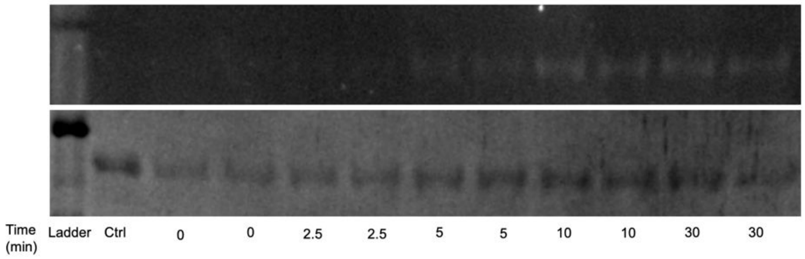

Figure S5. Results of the revised time course experiment for the reaction of GFP-biotin conjugate with Alexa Fluor 488 hydroxylamine purified with Ni-NTA prior to electrophoresis. SDS-PAGE with fluorescence imaging (top) and Coomassie blue staining (bottom) allowed reaction success and the protein content of each sample to be measured by densitometry using Image Lab.

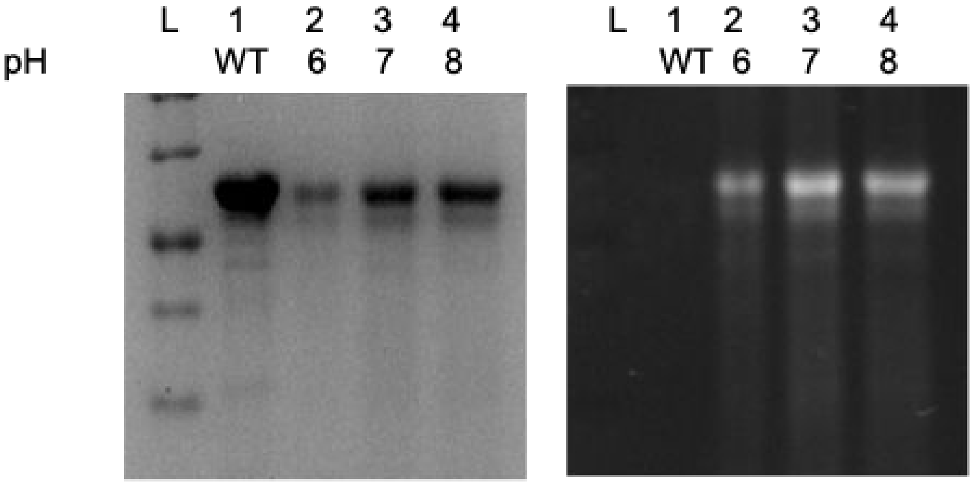

Figure S6. Bioconjugation optimization of pH. The GFP-biotin construct was buffer exchanged to pH 6, 7 and 8 and then incubated with aminooxy fluorophore for 1 hour. Fluorescence was observed under all 3 pH conditions, and the control reaction without the diyne exhibited no fluorescence when incubated with the aminooxy fluorophore.

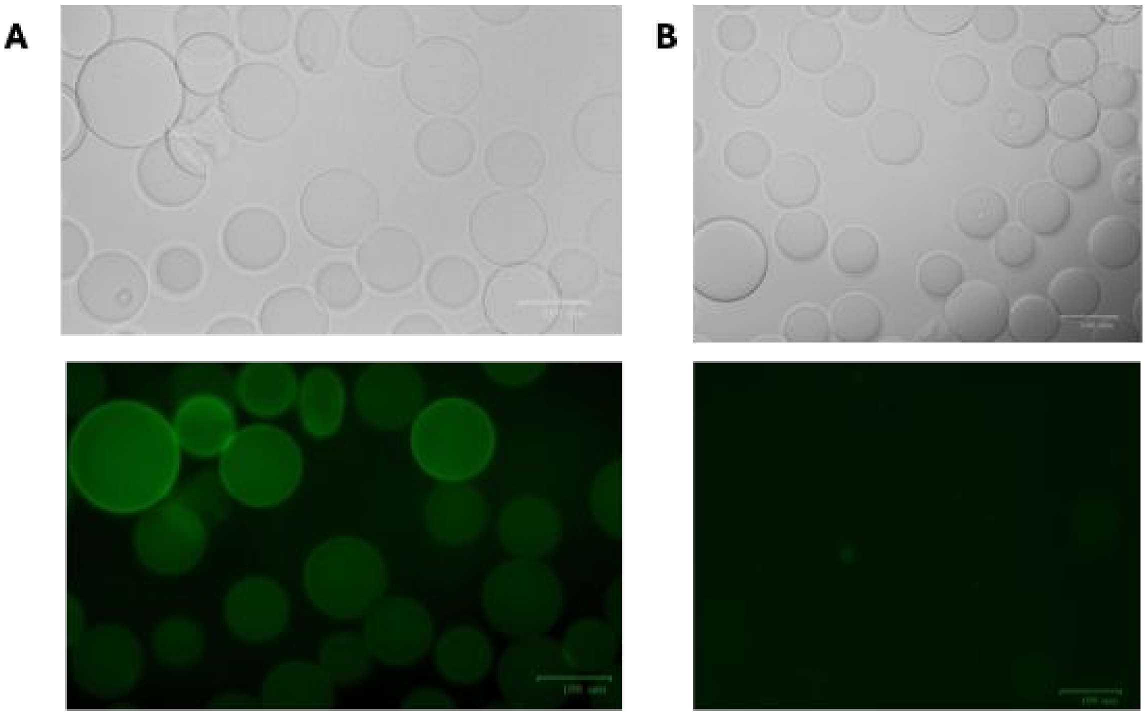

Figure S7. Further validation of trivalent conjugate preparation. To ensure that bead fluorescence was not due to any residual GFP fluorescence, ubiquitin (Ub) was expressed with *p*PrF at residue 48 and subjected to a Glaser-Hay coupling with biotin alkyne followed by an aminooxy coupling with aminooxy fluorophore. Following purification samples were incubated with streptavidin beads, washed, and imaged for the presence of fluorophore. A) Ub-biotin-fluorophore conjugate imaging with bright field image (top) and fluorescent image (bottom). Since ubiquitin is not fluorescent the only fluorescence is a result of the association of the aminooxy fluorophore with the biotin-protein. B) Control incubation with aminooxy fluorophore alone led to no observable fluorescence and no non-specific binding to the beads.

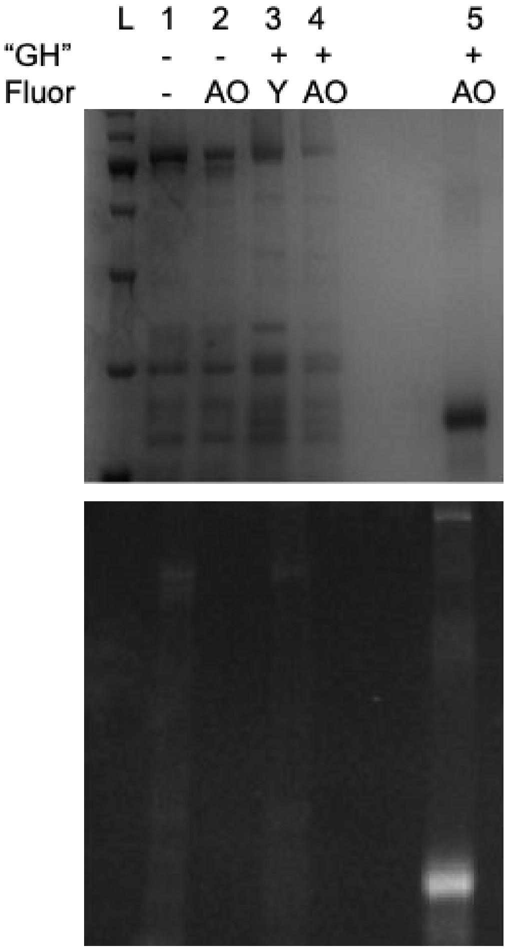

Figure S8. Assessment of undesirable side reactions. Cell lysate was treated under Glaser-Hay conditions, washed in molecular weight cut-off columns and then incubated with fluorophore. The lyate was then examined on SDS-PAGE for fluorescence that is not the result of the previously described cascade reaction as there are no alkynyl units in the cell lysate. Top Gel: Coomassie stain; Bottom Gel: Fluorescence image. Lane 1: cell lysate control not subjected to either Glaser-Hay conditions or fluorophore. Lane 2: cell lysate not subjected to Glaser Hay conditions, but incubated with aminooxy fluorophore, no fluorescence is observed. Lane 3: cell lysate subjected to Glaser-Hay conditions and incubated with alkyne fluorophore, no fluorescence is observed. Lane 4: cell lysate subjected to Glaser-Hay conditions and incubated with aminooxy fluorophore, no fluorescence was observed. Lane 5: positive control of GFP-*p*PrF-biotin conjugate incubated with aminooxy fluorophore, a fluorescent band is present. This suggests that no background oxidation of protein is occurring to generate aldehydes/ketones that could indirectly react with the aminooxy functionality.

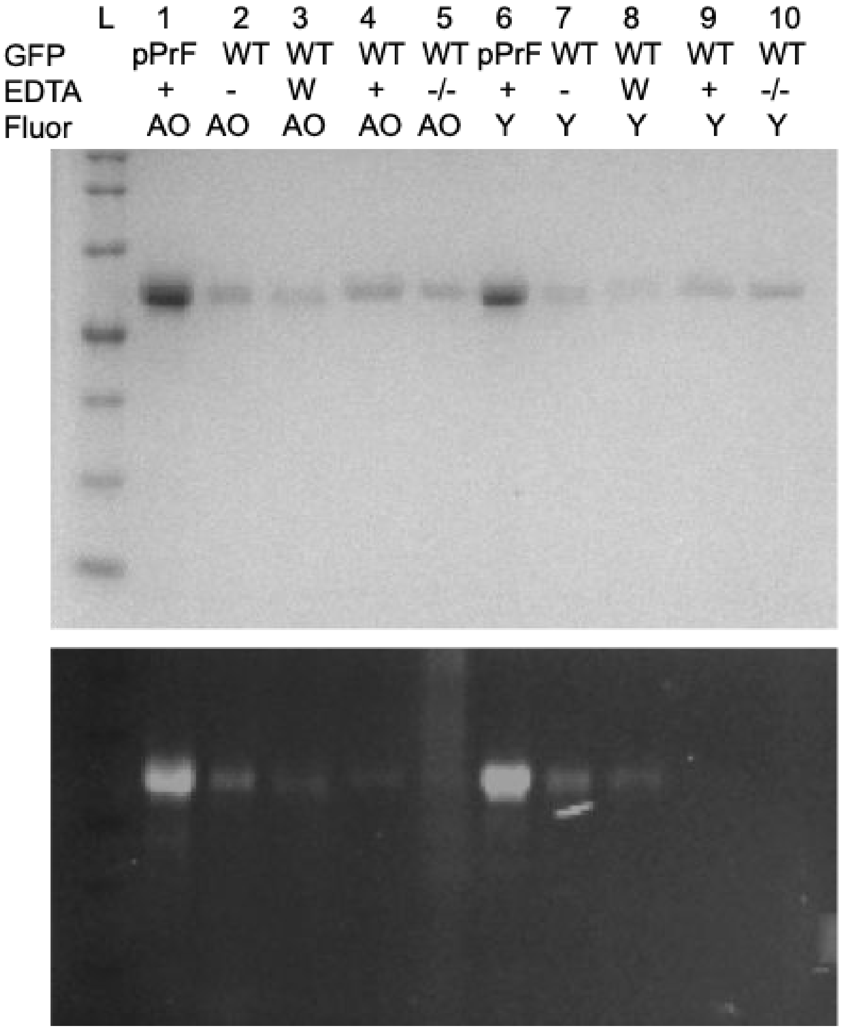

Figure S9. His Tagged Protein EDTA control. As some background fluorescence was observed with some His-tagged proteins, the reactions were washed with EDTA to determine if it was a result of non-specific binding of the copper to the His-tag followed by association with the fluorophore. Both the aminooxy fluorophore (AO) and the alkynyl fluorophore (Y) were examined. Lanes 1 and 6 represent positive controls where GFP-*p*PrF was either first Glaser-Hay coupled to biotin followed by incubation with aminooxy fluorophore (Lane 1) or GFP-*p*PrF was directly subjected to a Glaser-Hay coupling (Lane 6). Wild type GFP was then subjected to Glaser-Hay conditions followed by incubation with the respective fluorophore. In the absence of EDTA (Lanes 2 and 7) some background fluorescence is observed due to a non-specific interaction. However, washing (W) of reaction with EDTA (100 mM) significantly reduced the fluorescence (Lanes 3 and 8). If EDTA was added with the fluorophore no background fluorescence was observed (Lanes 4 and 9). In the absence of EDTA and Glaser-Hay conditions no fluorescence was observed (Lanes 5 and 10).

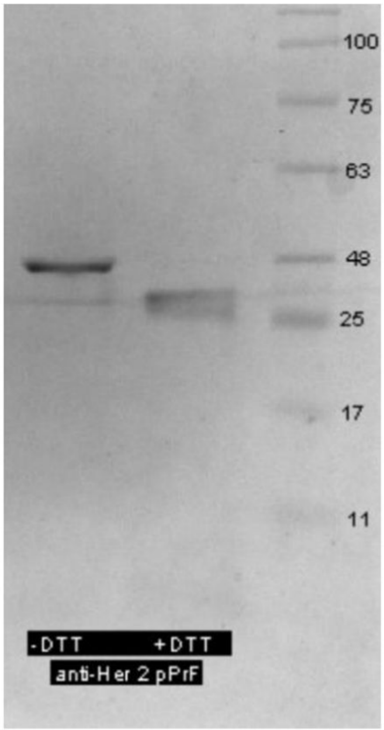

Figure S10. Non-reducing (left lane) SDS-PAGE revealed the presence of a band at ~50 kDa, confirming successful expression of anti-HER2-Fab-LC202-*p*PrF. Reducing SDS-PAGE (middle lane) resulted in the expected separation of the protein's heavy and light chains.

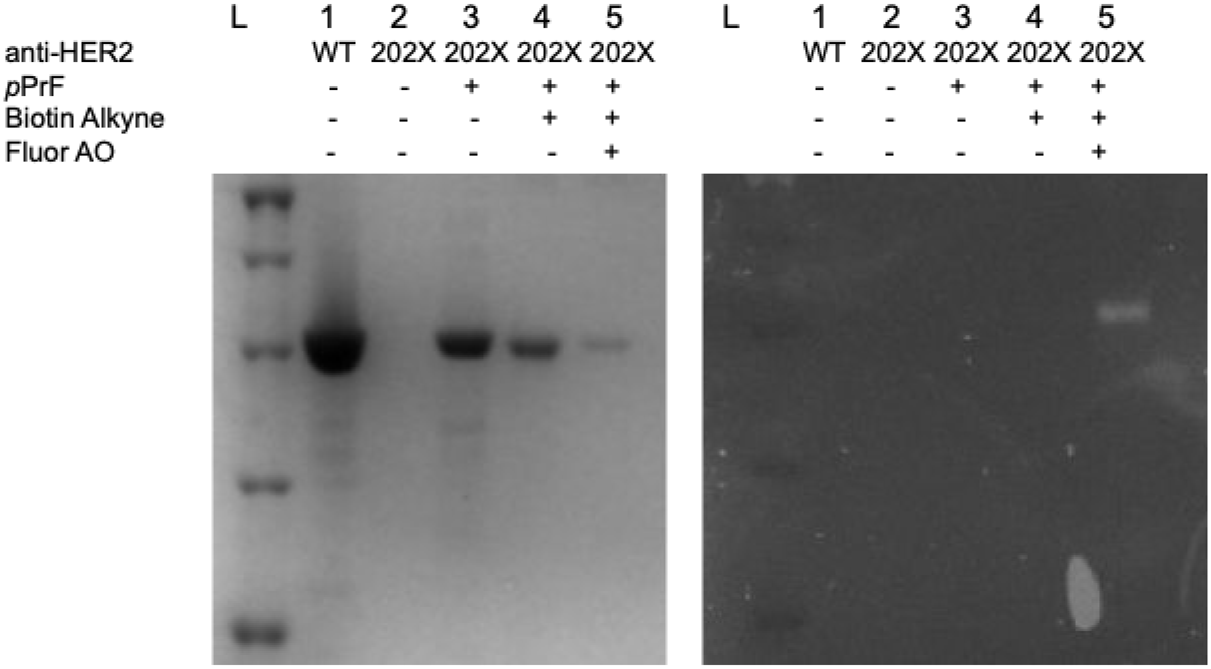

Figure S11. Full SDS-PAGE images of anti-HER2-Fab expressions and conjugations from Figure 4 of the manuscript.

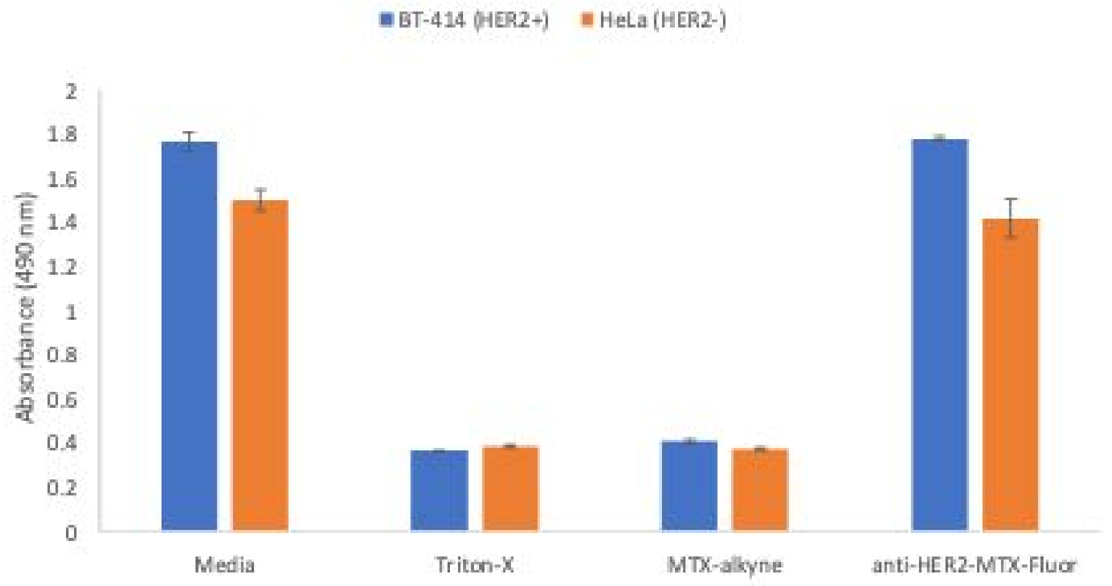

Figure S12. Cell viability assay with the MTX trivalent conjugate. Cell death was observed in the positive Triton-X control and with the MTX-alkyne indiscriminate of cell type (HER 2+/HER2-). No significant cell death was observed in the presence of the bioconjugate in either cell line relative to the media control.

A.

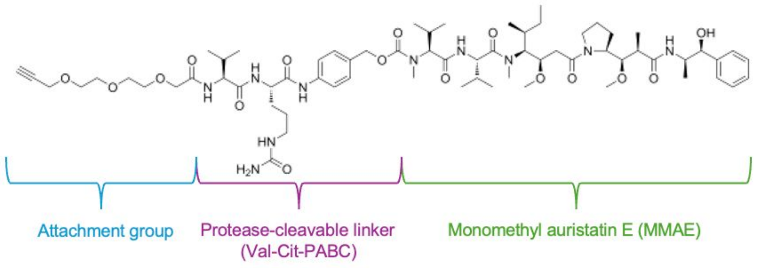

B

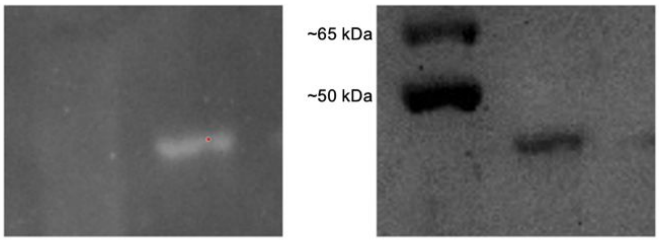

Figure S13. A) Structure of acetylene-linker-Val-Cit-PABC-MMAE (MMAE-alkyne). B) SDS-PAGE with fluorescence imaging (left) and Coomassie blue staining (right) confirmed the successful synthesis of the anti-HER2-Fab-MMAE-fluorophore conjugate (right lane). Once again, it was assumed that successful conjugation of the aminooxy fluorophore required successful synthesis of the anti-HER2-Fab-MMAE Glaser-Hay conjugate.

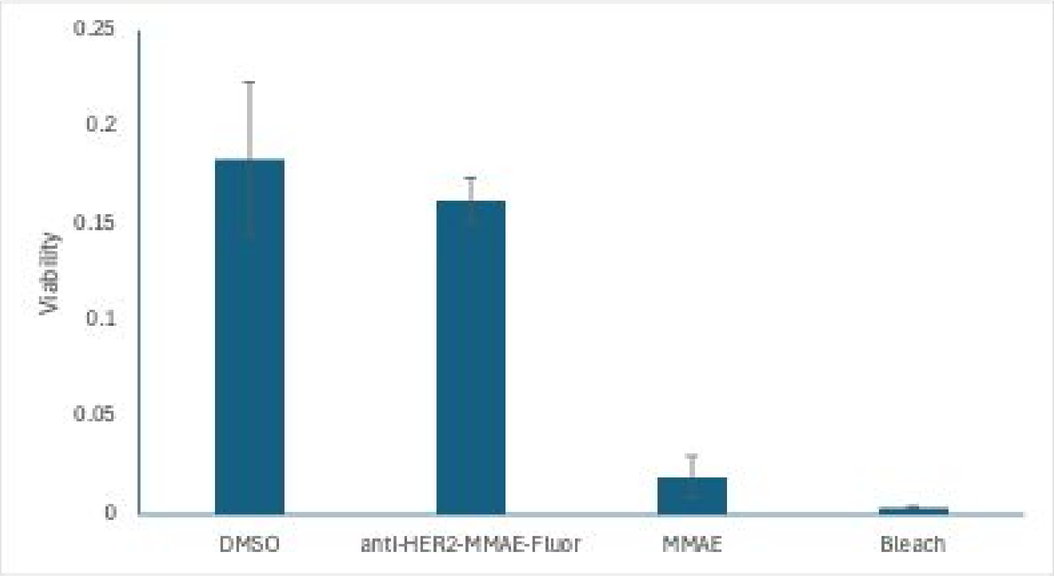

Figure S14. HeLa cell (HER2-) viability with trivalent conjugate. No significant decrease in viability was detected in an MTT assay between a DMSO control and the anti-HER2-Fab-MMAE-fluorophore conjugate. When treated with MMAE alone, cell viability significantly decreased in a comparable fashion to treating the cells with bleach. Experiments were performed in triplicate to determine standard deviations.
